# *Wolbachia* populations across organs of individual *Culex pipiens*: highly conserved intra-individual core pangenome with inter-individual polymorphisms

**DOI:** 10.1093/ismeco/ycae078

**Published:** 2024-06-11

**Authors:** Blandine Trouche, Hans Schrieke, Olivier Duron, A Murat Eren, Julie Reveillaud

**Affiliations:** IRD, MIVEGEC, University of Montpellier, INRAE, CNRS, 34394 Montpellier, France; IRD, MIVEGEC, University of Montpellier, INRAE, CNRS, 34394 Montpellier, France; IRD, MIVEGEC, University of Montpellier, INRAE, CNRS, 34394 Montpellier, France; Marine Biological Laboratory, Woods Hole, MA 02543, United States; Helmholtz Institute for Functional Marine Biodiversity at the University of Oldenburg, 26129 Oldenburg, Germany; IRD, MIVEGEC, University of Montpellier, INRAE, CNRS, 34394 Montpellier, France

**Keywords:** Wolbachia, mosquitoes, core pangenome, metapangenomics, subpopulations, single nucleotide polymorphism, single-nucleotide variants, punctual mutations

## Abstract

*Wolbachia* is a maternally inherited intracellular bacterium that infects a wide range of arthropods including mosquitoes. The endosymbiont is widely used in biocontrol strategies due to its capacity to modulate arthropod reproduction and limit pathogen transmission. *Wolbachia* infections in *Culex* spp. are generally assumed to be monoclonal but the potential presence of genetically distinct *Wolbachia* subpopulations within and between individual organs has not been investigated using whole genome sequencing. Here we reconstructed *Wolbachia* genomes from ovary and midgut metagenomes of single naturally infected *Culex pipiens* mosquitoes from Southern France to investigate patterns of intra- and inter-individual differences across mosquito organs. Our analyses revealed a remarkable degree of intra-individual conservancy among *Wolbachia* genomes from distinct organs of the same mosquito both at the level of gene presence–absence signal and single-nucleotide polymorphisms (SNPs). Yet, we identified several synonymous and non-synonymous substitutions between individuals, demonstrating the presence of some level of genomic heterogeneity among *Wolbachia* that infect the same *C. pipiens* field population. Overall, the absence of genetic heterogeneity within *Wolbachia* populations in a single individual confirms the presence of a dominant *Wolbachia* that is maintained under strong purifying forces of evolution.

## Introduction

*Wolbachia* is a maternally inherited intracellular bacterium widely used in biocontrol programs thanks to its ability to modulate the arthropod reproduction and to reduce the capacity to transmit pathogens [1–6] or the lifespan of pathogen host [7–13]. *Wolbachia* mainly infects the germline but also occurs in somatic tissues like fat body, hemolymph, central nervous system, for example [14]. It is mostly vertically transmitted through the female germline [15].

The endosymbiont induces multiple reproductive alterations to favor its spread by increasing the proportion of infected females (i.e. the transmitting sex) in the population: cytoplasmic incompatibility (CI) [15, 16], male killing [15–17], parthenogenesis [15, 16, 18], male feminization [15, 16]. CI is the most common reproductive manipulation and causes non-viable embryos when males infected with *Wolbachia* cross with uninfected females or when male and female are infected by incompatible *Wolbachia* variants [19–22]. In addition, transfection in *Aedes aegypti* mosquitoes with *Wolbachia* can diminish the transmission of some pathogens like Dengue, Chikungunya, or Zika [1–6]. Nevertheless, it can also enhance the transmission of others like West Nile virus [23]. Protective or reproductive phenotype disparities may be a result of species and strain-specific *Wolbachia*-host-virus interactions, which combination is also influenced by other factors like *Wolbachia* density, temperature, and host genetics, creating a system particularly difficult to disentangle.

Phylogenetic studies based on a multi-locus sequence typing (MLST) system comprised of conserved housekeeping genes [24] show that *Wolbachia* belong to at least 17 possible phylogenetic supergroups (named A-F, H-Q, and S), with the vast majority belonging to the group B-*Wolbachia* [16]. More recently, whole genome sequencing provided insights into the higher *Wolbachia* genetic diversity [16, 24–26]. In natural populations of the common house mosquito *Culex pipiens*, genotyping approaches using supplementary genes encoding proteins with ankyrin (ANK) motifs and Mobile Genetic Elements markers allowed to identify more than 100 genetically distinct *Wolbachia* variants belonging to five distinct phylogenetic groups (*w*PipI to *w*PipV) (referred to as *w*Pip strains [27–30]).

Nevertheless, most studies focusing on inter-individual variations of infections are based on a restricted set of genes that belong to the core and accessory genome, preventing comprehensive insights into the extent of homogeneity among *Wolbachia* cells within naturally infected individual hosts. *Wolbachia* are exposed to strong bottleneck effects during vertical transmission; they need to be transmitted to eggs, remain through embryogenesis, and finally become integrated in the founders of germ line stem cells [31] which may lead to monoclonal *Wolbachia* populations. These transmission bottlenecks are indeed shown to homogenize endosymbiotic bacteria in other systems like *Buchnera* in aphids, due to genetic drift and selection [32]. Although recently debated, the restricted niche of bacterial endosymbionts or pathogens also leads to the general assumption that only a few cells are sampled to start a new population from the millions of cells forming a within-host population [33]. Porter and Sullivan nevertheless note that *Wolbachia* could also follow a more indirect vertical transmission route by migrating from the somatic tissues to the germ line at each generation [31]. In addition, intra-individual variations of *Wolbachia* infections exist: *Wolbachia* can be horizontally transmitted [34, 35] and spread across distantly related arthropod taxa, a process that can generate co-infection of individual hosts by phylogenetically unrelated *Wolbachia* strains. Case studies include co-infection by the *w*AlbA and *w*AlbB *Wolbachia* strains in the invasive “Asian tiger” mosquito species *Aedes albopictus* [36, 37]. In addition to horizontal transfers, intra-individual structural variations of *Wolbachia* genomes have been shown. Chrostek and Teixeira for example showed intra-host variability in Octomom (a *Wolbachia* specific region including eight genes associated with density regulation) copy number between *Wolbachia* cells and within-host selection for faster replicating bacterial symbionts during the lifespan of flies [13, 38, 39]. Overall, gene and genome-level microbial population studies have been shedding light on cryptic bacterial microdiversity within single individual mosquitoes [40] or marine animals like mussels [41].

Despite the critical role of *Wolbachia* in biotechnological applications of pathogen transmission control strategies, whole genome-scale comprehensive insights into the extent of homogeneity within *Wolbachia* populations are lacking. Here, we used shotgun metagenomics to reconstruct *Wolbachia* genomes from single ovary and midgut samples obtained from adult *C. pipiens* mosquitoes collected in the South of France. We generated a pangenome to focus our analysis on the *Wolbachia* of *Culex* spp. core pangenome (genes present in single copy in reconstructed and reference genomes). We analyzed genetic variations within and between samples to investigate the putative presence of distinct *Wolbachia* populations in single individual organs and between individuals using a set of stringent filters to minimize the influence of bioinformatics artifacts.

## Materials and methods

### Sample collection, preparation, and sequencing

We collected and dissected individual mosquitoes, prepared four ovaries (O03, O07, O11, O12) and their corresponding midgut samples (M03, M07, M11, M12, together with two additional “orphan” samples M01, M09) for sequencing as in Reveillaud *et al.* [42] (see Supplementary Note S1 for further details).

### Metagenomic assembly and binning

We performed metagenomic analyses using anvi’o v7.1 [43, 44] and the metagenomic snakemake [45] workflow from the quality filtering to the merging of profile databases generated for each organ separately using the “anvi-run-workflow” program and the “--workflow metagenomics” flag. All the parameters used to set the snakemake workflows are written in the “config.json” files available in the Data Availability section. Although the ovaries raw reads have already been analyzed in Reveillaud *et al.* [42], we herein reanalyzed both ovaries and midguts raw reads following an exactly similar protocol to generate consistent analyses and comparable results for both organs. Briefly, during each workflow, we quality-filtered the raw reads from each sample using illumina-utils [46] v2.10 and the “iu-filter-quality-minoche” anvi’o program with default parameters. We assembled quality-filtered reads into contigs using MEGAHIT [47] v1.2.9, keeping only contigs with a length > 1000 nt. We performed read recruitment analyses by mapping the quality-filtered reads from all ovary samples onto the contigs of each ovary sample with “all against all” flag in config files using Bowtie2 [48] v2.3.5.1 and repeated the same procedure for midgut samples. We then used the “anvi-gen-contigs-database” program to generate anvi’o contigs databases for each individual assembly. This program computed k-mer frequencies for each contig, soft-split contigs with lengths > 20 000 bp into smaller ones, and identified ORFs in each contig using Prodigal [49] v2.6.3. We used the “anvi-run-hmms” program to identify HMM hits searching against the default HMM sources in anvi’o (Bacteria_71, Archaea_76, and Protista_83) and the “anvi-run-ncbi-cogs” program to assign functions to genes by searching their amino acid sequences against the COG20 [50] database using blastp [51] v2.10.1. We used the “anvi-profile” program to compute the coverage per nucleotide position and statistics for each metagenome assembly using the BAM files. We merged the resulting anvi’o profiles using the “anvi-merge” program. After the metagenomic snakemake workflow, we performed an automatic genome binning from assemblies using the “anvi-cluster-contigs” anvi’o program and the CONCOCT [52] algorithm (“--driver CONCOCT” flag) with a limited number of clusters (“--clusters X” flag) by sample (Supplementary Table S1) to separate bacterial and eukaryotic reads while avoiding bacterial genome dispersion (checked with “anvi-estimate-genome-completeness” program). Finally, we manually refined the bacterial bins obtained from each sample with the interactive program “anvi-refine.” In addition, we ran the “references-mode” of the metagenomic snakemake workflow to perform read recruitment of the quality-filtered reads from all samples to the refined *Wolbachia* Metagenome-Assembled Genome (MAG). We then removed low quality mappings with samtools [53] by filtering out reads with MAPQ <20, and finally performed anvi’o profiling and merged for inter-organ comparisons. Completion and redundancy of the five refined *Wolbachia* MAGs were estimated during anvi’o summary, as well as computed using the CheckM lineage workflow [54].

### Pangenomics

We performed pangenomic analysis for the five *Wolbachia* MAGs obtained andthree selected *Wolbachia* reference genomes: *w*PipPel isolated from *Culex quinquefasciatus* (NCBI Accession ID NC_010981.1) [55], *w*PipMol isolated from *Culex molestus* (NCBI Accession ID NZ_CTEH00000000.1) [56], and *w*PipJHB isolated from *C. quinquefasciatus* (NCBI Accession ID NZ_ABZA00000000.1) [57]. We downloaded the fasta files of the three selected *Wolbachia* reference genomes and reformatted them using the anvi’o “anvi-script-reformat-fasta” program. We then generated a new contigs database from the reformatted fasta files using the “anvi-gen-contigs-database” program. We identified HMM hits using the “anvi-run-hmms” program and used these hmm profiles to assign functions with “anvi-run-ncbi-cogs.” We created an external genome database including all these *Wolbachia* reference contigs databases. We created an internal genome database including the five *Wolbachia* MAGs contigs databases stored in the profile and contigs databases generated during the snakemake workflow with references mode. We then generated a genome storage database from both external and internal genome databases using the “anvi-gen-genomes-storage” program.

We computed the pangenome with the “anvi-pan-genome” program (using “--use-ncbi-blast” “--mcl-inflation 10” and the “genome-name” flags) and identified gene clusters for the five *Wolbachia* MAGs and three reference genomes based on amino acid sequence similarity. Only highly similar genes are added in a gene cluster during the anvi’o pangenomic workflow with almost no chance for two highly similar genes to end up in distinct gene clusters. We finally used the “anvi-display-pan” program to display the pangenome and visualize the distribution of gene clusters across genomes. From the pangenome summary, we obtained the id of gene clusters composed of genes occurring in single-copy in each genome. For convenience in the following analyses, we referred to genes belonging to these gene clusters as *Wolbachia* Single-copy Core Genes (*w*SCGs). Finally, we performed an additional sanity check on the selected *w*SCGs by confirming that their coverage was uniform over each metagenome, while the coverage of multi-copy gene clusters was variable and sparser (Supplementary Fig. S1).

### Prophage WO, MLST, wsp, cidA/B putative hits

We used the available results of blastn that identified *w*PipPel genes that match WO prophage regions (“WO_in_wPip_best_hit.txt” file from https://merenlab.org/data/wolbachia-plasmid/#identifying-genes-that-correspond-to-wo-prophages detailed in Reveillaud *et al.* [42]) together with the same custom R [58] script to identify gene cluster ids corresponding to these “phage like” gene calls.

We used blastn to identify MLST (MultiLocus Sequence Typing—*gatB*, *coxA*, *hcpA*, *ftsZ*, *fbpA*) and *wsp* genes from the PubMLST [59] (Public databases for molecular typing and microbial genome diversity) in *w*PipPel. Similarly, we identified best hits using blastn for *cidA* (NCBI Accession ID from MF444963 to MF444981 for the 18 *cidA* variants) and *cidB* (NCBI Accession ID from MF444982 to MF444996 for the 14 *cidB* variants) genes from Bonneau *et al*.[60]. Finally, we identified the gene clusters corresponding to these hits in *w*PipPel.

### Metapangenomics for inter-organ variability

We used a custom R script (based on https://merenlab.org/data/wolbachia-plasmid/#recovering-coverage-values-for-gene-clusters-of-the-wolbachia-pangenome-in-c-pipiens-metagenomes) to extract coverage values of metagenomes M11 and O11 mapped on MAG O11 and MAG M11 genes from the merged profile database and compute their means by gene cluster. We imported coverage values and WO prophage assignation described above to the pangenome database using the “anvi-import-misc-data” anvi’o program to build the metapangenome. We finally ran and edited the metapangenome using the “anvi-display-pangenome” program.

### Single nucleotide variants, single codon variants, single amino acid variants, and single nucleotide polymorphisms

We then used the “anvi-gen-variability-profile” program to extract the tables of Single Nucleotide Variants (SNVs, “--engine NT” parameter), Single Codon Variants (SCVs, “--engine CDN”), and Single Amino Acid Variants (SAAVs, “--engine AA”) from the anvi’o merged profile databases. Based on the summary from the pangenome, we added to these tables gene cluster information, including SCG/*w*SCG status and phage WO, MLST, *wsp*, and *cid*A/B putative assignation.

We quantified inter-sample variation by filtering the raw SNV tables, keeping between-sample SNVs occurring in *w*SCGs, not flagged as coverage outliers, and with a departure from the reference >0.98. These SNVs can be referred to as Single Nucleotide Polymorphisms (SNPs). We confirmed from the MAG coverage summaries that detection (or breadth of coverage) for the genes in which we found SNPs was equal to 1, to avoid partial mapping biases. We used the gene id and codon number information from the SNP tables, as well as the departure from reference >0.98 filter to obtain the associated SCV and SAAV tables from the raw tables.

We then focused our analysis on intra-sample variation by keeping only within-sample SNVs occurring in *w*SCGs, not flagged as coverage outliers (that can result from bioinformatic biases including breaks in or lack of assembly, unspecific mapping, etc.) and with entropy <0.2 and departure from consensus <0.2 (to discard those that could be due to sequencing errors and therefore considered as noise, https://merenlab.org/2013/11/04/oligotyping-best-practices/).

We visualized SNVs and SNPs through anvi’o with the « anvi-script-visualize-split-coverages » program and in Integrative Genome Viewer [61]. Finally, summary plots of the data contained in SNV and SNP tables were obtained in R. The fully reproducible workflow for this analysis is available at https://github.com/jreveillaud/Wolbachia-subpopulations.

## Results

### Reconstruction of *Wolbachia* MAGs in one midgut and four ovaries of *C. pipiens* individuals

Our quality filtering of raw reads sequenced from midgut and ovaries samples from individual mosquitoes resulted in 94 024 472 and 75 040 983 paired-end reads on average, respectively (Supplementary Table S2). Individual sample metagenomic assembly generated on average 166 820 contigs >1 kb recruiting between 24% and 92% of filtered reads (Supplementary Table S2). To estimate the proportion of eukaryotic reads (that we herein refer to as “contamination” in opposition to bacterial reads) in our metagenomes, we used phyloFlash [62] to annotate short reads based on the SILVA rRNA database [63] (see Supplementary Note S2 for further details). Results suggested that the vast majority of our reads (over 99% for each sample) originated from eukaryotic organisms, especially in midgut metagenomes (Supplementary Fig. S2; Supplementary Table S3).

Despite the high eukaryotic contamination rate, we reconstructed *Wolbachia* genomes from all ovary metagenomes and one of the four midgut metagenomes (M11) with 91.5% completion and 0% redundancy estimated based on Bacterial Single-Copy core Genes (BSCGs) from the collection of Campbell *et al*. [64] after manual refinement (Table 1, Supplementary Table S1). This is, to our knowledge, the first *Wolbachia* draft genome reconstructed from a *Culex* mosquito midgut. Of note, midgut metagenomes M01 and M09, which had no corresponding ovary samples, were solely used to improve binning (by providing additional differential coverage information). These samples were not further investigated as we did not reconstruct bacterial genomes from them. During the final read recruitment step, the refined MAGs recruited between 0.83% and 3.48% of reads in the metagenomes they were respectively reconstructed from.

**Table 1:** Refined *Wolbachia* MAGs estimates including completion and redundancy rates, number of contigs, total number of nucleotides (length), and GC content.

***Wolbachia* MAG**	**Completion (%) (BSCGs)**	**Redundancy (%) (BSCGs)**	**Completion (%) (CheckM)**	**Redundancy (%) (CheckM)**	**Number of contigs**	**Length (bp)**	**GC content (%)**
M11	91.55	0	100	0.09	138	1 331 260	34.2
O11	91.55	0	100	0.09	119	1 290 070	34.2
O03	91.55	0	99.15	0.09	73	1 164 954	33.8
O07	91.55	0	100	0.09	143	1 340 038	34.4
O12	91.55	0	99.15	0.09	75	1 181 440	33.9

The MAGs showed a high completion >90% and no redundancy based on the use of Bacterial Single-Copy core Genes (BSCGs) from the collection of Campbell et al. [64] and the use of CheckM [54].

### Comparison of *Wolbachia* MAGs between organs of the same individual

As we reconstructed for the first time a *Wolbachia* MAG from a midgut metagenome, we investigated the putative occurrence of organ-specific gene clusters at the individual level. We first observed 19 gene clusters that seemed to be unique to *Wolbachia* MAG M11 and four gene clusters possibly unique to *Wolbachia* MAG O11 (Supplementary Fig. S3; Supplementary Table S4). Nevertheless, in our metapangenomic analysis, the mapping of quality filtered reads onto the two *Wolbachia* MAGs showed that those gene clusters had coverage in all samples and thus were not unique to one specific *Wolbachia* MAG (Supplementary Fig. S3). The absence of some genes in our *Wolbachia* MAGs could be explained by assembly breaks and/or the exclusion of contigs with length < 1000 bp. We therefore did not observe clear evidence of organ-specific *Wolbachia* populations (Supplementary Table S4; Supplementary Fig. S3).

### Single-copy core genes in *Wolbachia* MAGs and reference genomes

Furthermore, we compared gene content between our five newly reconstructed *Wolbachia* MAGs and three *Wolbachia* reference genomes (*w*PipPel, *w*PipMol, and *w*PipJHB) through pangenomic analyses. Overall, we identified 1205 gene clusters (Supplementary Table S5), among which 890 were single-copy gene clusters, i.e. composed of a single gene sequence for each *Wolbachia* MAG and reference genome. The sequences belonging to these single-copy gene clusters were referred to as *Wolbachia* Single-copy Core Genes (*w*SCGs) within each MAG (Supplementary Table S6) as discussed in the Material and Methods section. To note, we identified *w*SCGs corresponding to MLST and *wsp* genes but not to any *cid* gene (Supplementary Table S6).

### *Wolbachia* population genetics between individual mosquitoes (inter-sample variability)

We looked for SNPs, i.e. variable positions with 100% divergence from the reference sequence, to investigate the possible presence of fixed mutations between individuals within *w*SCGs. After recruiting reads from all metagenomes to the five reconstructed MAGs, we filtered our mapping results to only keep reads with a mapping quality over 20. This removed between 1.13% and 2.92% of recruited reads, losing some information but increasing the robustness of our analysis (Supplementary Fig. S4). We then selected point mutations in *w*SCGs with departure from reference over 0.98, always making sure that they were not identified as coverage outliers. Finally, we checked that detection (or breadth of coverage) was equal to 1 on the considered genes to avoid errors due to partial recruitment.

**Figure 1:**
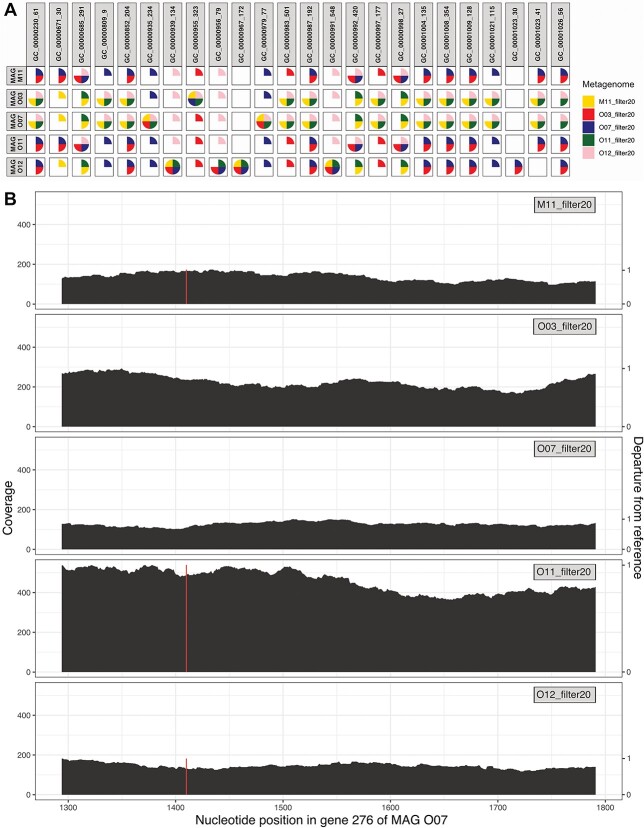
SNPs identification and visualization; (A) representation of the variable positions (vertical name corresponding to gene cluster id + codon id in which it occurs) in the five *Wolbachia* MAGs and the metagenomes in which a SNP is identified (colored pie chart); (B) visualization of gene 276 (gene cluster GC_00001009) from contig 124 766 in MAG O07; a SNP is identified in metagenomes M11, O11, and O12, in first position in codon 128 (represented by a contrasting bar); this gene was annotated as « Holliday junction resolvasome RuvABC endonuclease subunit RuvC » by COG20; additional visualizations of SNPs are available in Supplementary Figs S5–S7.

We identified SNPs for all inter-individual comparisons, except when mapping metagenome M11 to *Wolbachia* MAG reconstructed from O11, and vice-versa (Supplementary Tables S7 and S8). In total, SNPs were identified in 23 gene clusters, with 22 variable positions in MAGs M11, O03, O07, O11 and 23 in MAG O12 (Fig. 1A; Supplementary Table S9). All SNPs gave rise to SCVs (Supplementary Table S10) and a number of them resulted in SAAVs (Supplementary Table S11), with a mean SAAV to SCV ratio between 0.75 and 0.82 (Supplementary Table S12).

### *Wolbachia* population genetics within individual mosquitoes (intra-sample variability)

In contrast to the small number of inter-individual SNPs across genomes we described above, the raw SNVs from metagenomic read recruitment results suggested a remarkable number of intra-individual variants. However, a higher level of scrutiny revealed that these variants in the core pangenome could be attributed to bioinformatic artifacts (See Supplementary Note S3, Supplementary Figs S8–14 and Table S13 for further details of the analysis and visualizations).

## Discussion

We reconstructed *Wolbachia* MAGs from both the ovaries and midgut of one *C. pipiens* individual for the first time, as well as from the ovaries of three additional *C. pipiens* specimen using metagenomic approaches. Our metapangenomic analyses indicated that ovaries and midgut from a single mosquito share similar *Wolbachia* gene content, suggesting the uniform segregation and the lack of strain selection across organs. In addition, variability analyses at the inter-sample level showed the existence of synonymous and non-synonymous SNPs, with different occurrence patterns across individuals, suggesting fixed punctual mutations and multiple *Wolbachia* populations. However, a detailed SNV investigation within *w*SCG at the intra-sample level showed the absence of punctual mutations.

Globally, *Wolbachia* is manipulated with the idea that it is mono-clonal in transfection and naturally infected mosquito specimen. *Wolbachia* is predominantly extracted from egg cytoplasm of an infected species before being transferred to a recipient one [65] during transfection. Although a relative stability of *Wolbachia* genomes has been observed following the artificial transfer of the bacterium between host species for several years, higher mutation rates were recently shown in *A. aegypti* cell lines, suggesting that different population dynamics can occur following distinct selective pressures within specific environments [66]. Similarly, the action of selective sweep has been documented on *Wolbachia* genomes from *Drosophila melanogaster* [67]*.* The absence of genetic heterogeneity shown here in the *Wolbachia* core pangenome within single naturally-infected mosquito organs and specimen is congruent with evolutionary processes acting against mutations within samples, including reproductive bottleneck and a strong purifying selection. In addition, we did not detect different gene content nor any SNPs in *Wolbachia* from different organs of the same individual, highlighting the uniformity of *Wolbachia* at the mosquito level. Our data agree with a single *Wolbachia* population that is transferred from the mother to the offspring [14, 68] and then from the germ line to the somatic tissue.

The observation of SNPs, differentially co-occurring across individuals, and in some cases non-synonymous, nevertheless question the emergence and evolution of variants. As of now, the evolutionary processes giving rise to these fixed mutations remain unknown. Theoretically, non-neutral processes could drive the emergence of distinct variants conferring evolutionary advantages to their host, such as protection against pathogens in tripartite *Wolbachia*-host-pathogen interactions [69]. However, it could just as likely result from drift and fixation in the progeny through a random transmission event. These processes would be studied most efficiently by monitoring *Wolbachia* evolution in the progeny of an iso-female line over a long period of time.

Despite the first striking identification of SNVs in *w*SCG genes within samples (Supplementary Table S7), a close examination of SNVs and coverage variations highlighted cryptic and hidden bioinformatic bias, most likely due to the fragmented nature of *Wolbachia* MAGs. Indeed, although we focused our analysis on SNVs occurring only within *w*SCG (that showed a single copy gene signature using a combined pangenomic and metapangenomic approach), an in-depth investigation revealed the occurrence of SNVs significantly correlated with a subtle increase in coverage. Blast outputs confirmed these bioinformatic artefacts, suggesting these data were due to (i) hidden conserved domains within target genes, as well as (ii) genes that were not reconstructed in fragmented *Wolbachia* genomes despite high completion values (91.5% to 100% depending on anvi’o or CheckM estimates). Indeed, a high number of transposable elements render the obtention of circular *Wolbachia* genomes particularly challenging [70]. In addition, ANK repeat domain encoding genes, particularly numerous in *Wolbachia* genomes (23 in *w*Mel, 60 in *w*Pip strain [71]), could impede assembly and consequently favor non-specific read recruitment. Similar patterns of unspecific read recruitment could be observed for other intracellular bacteria including pathogens like *Ehrlichia*, which shows a high number of tandem repeats [72].

Overall, making good use of *Wolbachia* requires information on the genetic variation of the host, the pathogen, and the endosymbiont at fine scale, as distinct variants can alter pathogen virulence as well as the efficiency of the protective or reproductive phenotype. *Wolbachia* is widely used in antivectorial programs worldwide to fight diseases, and knowledge of bacterial diversity within and between single individuals is critical. Here our analysis focused on the core pangenome of *Wolbachia* due to the type of data we were working with (short read). It would be beneficial to extend it to the whole genome using other techniques such as long-read sequencing that could yield less fragmented genomes and allow studying structural variations at the individual scale.

## Supplementary Material

2024_04_25_Supplementary_Information_with_better_figures_ycae078

Table_S1_MAG_binning_ycae078

Table_S2_read_recruitment_stats_ycae078

Table_S3_phyloflash_results_ycae078

Table_S4_metapangenome_ycae078

Table_S5_pangenome_GCs_ycae078

Table_S6_GCs_wSCG_WO_ycae078

Table_S7_raw_SNV_table_with_SCG_ycae078

Table_S8_SNP_table_ycae078

Table_S9_all_positions_with_SNPs_ycae078

Table_S10_SCV_table_ycae078

Table_S11_SAAV_table_ycae078

Table_S12_summary_SNP_SAAV_ratio_ycae078

Table_S13_retained_SNVs_ycae078

## Data Availability

The raw sequencing data for the shotgun metagenomes of midgut and ovary samples are available in the European Nucleotide Archive via accession number PRJEB56379 and PRJEB26028, respectively. In addition, we made available the merged anvi’o profiles for the midgut and ovary metagenomes (https://doi.org/10.5281/zenodo.7183277), the FASTA files for the five *Wolbachia* MAGs (https://doi.org/10.5281/zenodo.7183303), the anvi’o merged profile databases for the *Wolbachia* MAGs used for the SNVs and metapangenomic analyses (https://doi.org/10.5281/zenodo.7183324). A reproducible bioinformatics workflow including scripts used for all computational analyses is available at the URL https://github.com/jreveillaud/Wolbachia-subpopulations (https://doi.org/10.5281/zenodo.11059970).
