# Supplementary material for: Wolbachia populations across organs of individual Culex pipiens: highly conserved intra-individual core pangenome with inter-individual polymorphisms
Source: ISME Commun. 2024 Jun 11;4(1):ycae078. doi: 10.1093/ismeco/ycae078 (PMC11195471; doi:10.1093/ismeco/ycae078)
Supplement: 2024_04_25_Supplementary_Information_with_better_figures_ycae078 [file 2024_04_25_supplementary_information_with_better_figures_ycae078.pdf]

***Wolbachia* populations across organs of individual *Culex pipiens*: highly conserved intra-individual core pangenome with inter-individual polymorphisms**

Supplementary Notes

Supplementary Note 1 – Sample collection, preparation, and sequencing.

Briefly, we collected *Culex* mosquito specimens in Languedoc (France) in May 2017 using a carbon dioxide mosquito trap (Camping l'Europe de Vic La Gardiole, Entente Interdépartementale de Démoustication EID, Méditerranée). We transported them alive to the laboratory directly afterward and anesthetized females by incubating them for 4 minutes (min) at -20°C. We washed them with ethanol 96% during 1 min in order to remove eventual surface contaminants and PBS 1X to avoid DNA precipitation due to ethanol. We transferred them onto a sterile microscope slide with one drop of sterile PBS 1X and dissected ovaries and midgut from each mosquito using sterilized tweezers. Of note, we thoroughly flame-sterilized dissecting tweezers and needles between each individual and organ dissection to avoid any transfer of biological material from one sample to another.

Finally, we stored four ovaries (O03, O07, O11, O12) and their corresponding midgut samples (M03, M07, M11, M12, together with two additional "orphan" samples M01, M09) from individual mosquitoes at -80°C to preserve them until further processing. We extracted the DNA from each sample using the MoBio PowerFecal DNA Isolation Kit (QIAGEN Inc., Germantown, MD, USA). We sonicated 3.8-5.7 ng of genomic DNA using an E200 Covaris instrument (Covaris, Woburn, MA, USA). We used the NEBNext Ultra II DNA Library Prep Kit for Illumina (New England Biolabs, Ipswich, MA, USA) to end-repair and 3'-adenylate the resulting fragments and added NEXTflex PCR-free barcode adapters (Bioo Scientific, Austin, TX, USA). We purified the ligation products using Ampure XP (Beckman Coulter, Brea, CA, USA) and amplified the DNA fragments by PCR (>200 bp; 2 PCR reactions, 14 cycles) using Illumina adapter-specific primers and NEBNext Ultra II Q5 Master Mix (NEB). We used an Agilent 2100 Bioanalyzer (Agilent Technologies, Santa Clara, CA, USA) to analyze library

profiles and performed a qPCR analysis using the KAPA Library Quantification Kit for Illumina Libraries (KapaBiosystems, Wilmington, MA, USA). We sequenced the library using a HiSeq4000 Illumina sequencer (Illumina, San Diego, CA, USA) at the Genoscope in Evry, France, generating 151 bp paired-end reads. Finally, we used a cluster intensity and chastity filter as described in [1] in order to filter the raw sequencing.

Supplementary Note 2 – Estimation of host contamination.

We estimated the host contamination in the ovary and midgut quality filtered short reads using the software phyloFlash [2] v3.4. We used the "phyloFlash.pl" script with the "--readlength 150" and "--almosteverything" flags that i) extracted the small-subunit rRNA (SSU rRNA) sequences from our data by mapping our reads to the SILVA SSU Ref database [3], ii) assembled the extracted reads into SSU rRNA using SPAdes [4] assembler, iii) estimated the proportion of assembled sequences by re-mapping them to the full-length SSU sequences, iv) provided a taxonomic summary of the reads from the initial mapping, the full-length SSU rRNA sequences and the unassembled sequences. We plotted the taxonomic assignment of the mapped reads on the SILVA SSU databases using a custom R script (R version 3.6.3 [5]) to visualize the estimation of the host contamination in our samples.

Supplementary Note 3 – *Wolbachia* SNVs at the intra-sample level.

1. Identification of Single Nucleotide Variants (SNVs) in individual mosquitoes

We mapped the metagenomic reads from each sample to the corresponding *Wolbachia* MAG to access intra-sample variability, and filtered out reads mapping with a quality lower than 20 (Figure S4). We characterized a mean of 2,036 raw SNVs within our *Wolbachia* MAGs (intra-sample variability) with a mean coverage of 252X (Table S7). After our filtration steps (entropy > 0.2 and departure from consensus > 0.2), we obtained a final number of 716 SNVs on average with a mean coverage value of 181X in our samples (Table S7). In order to quantify the extent of variability at variable nucleotide positions in the metagenome compared to the reference sequence, we used the metric ‘departure from consensus’, which reports the ratio of nucleotides at a given position that do not match to the consensus nucleotide. The departure from consensus

values varied from 0.2 to 0.74 indicating between 20 to 74% of base variation at one SNV position in comparison to the consensus nucleotide within each metagenome (Table S7).

We then focused our analysis on SNVs occurring within gene calls in order to study the potential influence of genomic variation on protein-coding genes in our MAGs. A SNV located in the 1st or 2nd position of the codon will be more susceptible to result in a non-synonymous mutation (resulting in changes in the amino-acid sequence) whereas a SNV located in the 3rd position will be more likely to result in a synonymous mutation (no changes in the amino-acid sequence). We also removed coverage outliers. We reported 107, 74, 42, 152 and 35 SNVs occurring in gene calls for MAGs O11, M11, O03, O07 and O12, respectively (Figure S8, Table S7). Among those gene-call SNVs, we counted a mean number of 22, 18 and 42 SNVs at the 1st, the 2nd and the 3rd base position, respectively (Table S7).

Focusing our analysis on the variable positions falling within *w*SCGs, after filtering out coverage outliers and low departure SNVs, we detected a mean of 3.2 SNVs in 1 gene for each MAG (Figure S8; Table S7). Interestingly, while single-copy gene clusters accounted for 73.86% of all gene clusters in the pangenome, only a limited number of SNVs were detected within the corresponding *w*SCGs (17.3% for MAG M11, 29.27% for O03, 3.11% for O07, 19.97% for O11, 39.17% for O12) (Figure S8).

In *Wolbachia* MAG M11, we reported 2 SNVs falling in one *w*SCG (gene caller id 301) that was not annotated to any function using COG20 and KEGG databases. In *w*SCG 301, 1 SNV falls at the 2nd position in codon and 1 SNV at the 3rd position in codon. On average, these SNVs have a coverage of 189X and a departure from consensus of 0.21 (Table S7, S13).

In *Wolbachia* MAG O11, we reported a total of 7 filtered SNVs in a *w*SCG (gene caller id 755) annotated to an « Ankyrin repeat » function by the COG20 database. In *w*SCG 755, respectively 2, 3 and 2 SNVs fall at the 1st, 2nd and 3rd position in codon, respectively. On average, these SNVs have a coverage of 486X and a departure from consensus of 0.39 (Table S7, S13).

In *Wolbachia* MAG O03, we reported 2 filtered SNVs in 1 *w*SCG (gene caller ids 589) annotated to an « ATP-dependent 26S proteasome regulatory subunit » (COG20), falling in 1st and 2nd positions in codon. On average, these SNVs have a coverage of 246X and a departure from consensus of 0.22. (Table S7, S13).

In *Wolbachia* MAG O07, we reported 1 filtered SNV in one *w*SCG (gene caller id 230), with no assigned function. This unique SNV falls at the 2nd position in codon, has a coverage of 146X and a departure from consensus of 0.22 (Table S7, S13).

In *Wolbachia* MAG O12, we reported a total of 4 filtered SNVs in 2 *w*SCGs (gene caller ids 423 and 640). In *w*SCG 423, with no annotated function, 1 SNV falls at the 1st position in codon and 1 SNV at the 3rd position in codon. In *w*SCG 640, annotated to an « ATP-dependent 26S proteasome regulatory subunit » (COG20), 1 SNV falls at the 1st position in codon and 1 SNV at the 2nd position in codon. On average, these SNVs have a coverage of 194X and a departure from consensus of 0.25 (Table S7, S13).

2. *Visual inspection of intra-individual SNVs*

We visualized the retained SNVs in the context of the gene where they appear (Figure S9-14). A close examination of SNVs in gene 640 (annotated to an "ATP-dependent 26S proteasome regulatory subunit" by COG20 database, Figure S9) from split 99075 of *Wolbachia* MAG O12 showed a concomitant increase of SNV departure from consensus and coverage values for gene 640 (Figure S9A-C) despite having a single copy gene signature. A significant correlation was confirmed between departure from consensus and coverage values ($r = 0,66$; $p = 2.10^{-4}$; Figure S9D). In addition, blasting a selected sequence from *w*SCG 640 in MAG O12 (contig 99075, nucleotides 16,898 to 17,082, left-hand side in Figure S9A-C) against the full MAG O12 with a discontiguous megablast (to detect less similar sequences) revealed a hit with a perfect match together with one additional hit with lower identity (73.72% over 74% of the query).
Similar visualizations were produced for all 6 genes considered (Figure S9-14). In all cases, the detection of SNVs was linked to a variation in coverage, which produced a « variable » region, though most of the raw SNVs were filtered out either due to a departure from consensus below the fixed threshold or to being coverage outliers. These data highlight non-specific read recruitment that are likely due to i) hidden repeated domains within the whole MAG ii) genes not reconstructed in fragmented genomes.

Supplementary Tables

Supplementary Table 1 – *Wolbachia* MAGs estimates. Number of clusters used for binning (using the “--clusters” flag of the “anvi-cluster-contigs” anvi’o program as detailed in Material and Methods section), raw percent completion (before refinement), raw percent redundancy (before refinement), percent completion (after refinement), percent redundancy (after refinement), number of contigs (after refinement), length (after refinement), GC content (after refinement).

Supplementary Table 2 – Quality filtering and assembly statistics for each sample (M01, M03, M07, M09, M11, M12, O11, O03, O07, O12). Total number of raw pairs of reads, number of filtered pairs of reads, percent of filtered (passed) reads, number of contigs > 1kb, L50, N50 and percent of high-quality recruited reads by sample.

Supplementary Table 3 – Taxonomic profiling of metagenomes. Taxonomic summaries based on the mapping hits of the quality filtered reads to the SILVA SSU rRNA database performed during the phyloFlash analyses for each sample (M01, M03, M07, M09, M11, M12, O03, O07, O11, O12). The table reports all the identified taxa (from the Domain to the Species rank) and their number of reads.

Supplementary Table 4 – Specimen 11 inter-organ *Wolbachia* pangenomic analysis. Gene cluster table from the pangenomic analysis performed on *Wolbachia* MAGs M11, O11 and the *w*PipPel reference genome. The table reports for each identified gene cluster: ids (unique_id, gene_cluster_id), bin and genome names (bin_name, genome_name), gene caller id (gene_callers_id), number of genomes where gene cluster occurs (num_genomes_gene_cluster_has_hits), number of genes in gene cluster (num_genes_in_gene_cluster), maximum number of paralogs (max_num_paralogs), identification as Single-Copy Core genes (SCG), functional, geometric and combined homogeneity indices (functional_homogeneity_index, geometric_homogeneity_index, combined_homogeneity_index), KEGG assignation information (KEGG_Module_ACC, KEGG_Module, , KEGG_Class_ACC, KEGG_Class), COG20 assignation information (COG20_PATHWAY_ACC, COG20_PATHWAY, COG20_FUNCTION_ACC,

COG20_FUNCTION, COG20_CATEGORY_ACC, COG20_CATEGORY), KOfam assignation information (KOfam_ACC, KOfam, KEGG_Module_ACC) and corresponding amino acid sequence (aa_sequence).

Supplementary Table 5 – Gene cluster statistics. Gene clusters identified during the pangenomic analysis performed on all reconstructed *Wolbachia* MAGs (MAGs M11, O11, O03, O07 and O12) and the three selected *Wolbachia* reference genomes *w*PipPel, *w*PipMol, *w*PipJHB. The table reports the same information as in Supplementary Table 4.

Supplementary Table 6 – Single-copy Core Genes from *Wolbachia*. *w*SCGs identified among the unique GCs computed during the pangenomic analysis performed on all reconstructed *Wolbachia* MAGs and selected *Wolbachia* reference genomes. Table reports for each unique GC: gene cluster id (gene_cluster_id), *w*SCG info (1=*w*SCG), prophage WO regions assignment (WO_assignmnent, WO_pct_alignment), *cid* genes assignment (*cid*_genes_from_Bonneau_et_al._2018 [6], *cid*_Bonneau_pct_alignment), and the MLST and *wsp* genes assignment (MLST_and_*wsp*_genes_from_PubMLST [7], MLST_*wsp*_pct_alignment).

Supplementary Table 7 – Raw SNV tables. Table reports for each SNV position: unique identifier of the SNV (entry_id), unique position identifier of the SNV (unique_pos_identifier), nucleotide position of the SNV in the split and the contig (pos, pos_in_contig), name of the split where SNV is occurring (split_name), sample where the SNV has been identified (sample_id), unique gene caller identifier where the SNV is occurring (corresponding_gene_call with "-1" if the position is not in a gene call), coding or non-coding status of the gene (in_noncoding_gene_call, in_coding_gene_call), nucleotide position in codon (base_pos_in_codon), order of the codon in the gene call (codon_order_in_gene with "-1" if the position is not in a gene call, codon_number), gene length (gene_length), number of recruited reads mapping to this position (coverage), outlier status of the SNV based on coverage estimated in split and contig (cov_outlier_in_split, cov_outlier_in_contig), number of mapped reads covering the position corresponding to the different bases (A, C, G, N, T), reference nucleotide in the reference genome (reference), most mapped nucleotide at the position (consensus), the two most represented nucleotides at the position (competing_nts), ratio of nucleotides in a given position that diverge from the reference nucleotide (departure_from_reference), ratio of nucleotides in a given position that diverge from the

consensus nucleotide (departure_from_consensus), ratio of the second most frequent nucleotide to the consensus nucleotide (n2n1ratio) and the entropy value (entropy). All these SNV information are also explained at the following link https://merenlab.org/2015/07/20/analyzing-variability/. Moreover, the table reports information related to gene clusters where the SNV falls in as in Table S6 (including the gene cluster id and the *w*SCG status of a gene) but also the prophage WO regions assignment (WO_assignmnent, WO_pct_alignment), *cid* genes assignment on the gene where the SNV occurred (*cid*_genes_from_Bonneau_et_al._2018 [6], *cid*_Bonneau_pct_alignment), the MLST and *wsp* genes assignment (MLST_and_*wsp*_genes_from_PubMLST [7], MLST_*wsp*_pct_alignment).

Supplementary Table 8 – Filtered SNP table (at the inter-sample level). Information is reported as in Table S7 for the SNVs identified as Single Nucleotide Polymorphisms (SNP), i.e. within *w*SCGs, with departure from reference over 0.98, and not identified as coverage outliers.

Supplementary Table 9 – Summary of SNP positions in the five *Wolbachia* MAGs, grouped by gene cluster. Gene clusters were identified as in Table S5, grouping one gene from each MAG. The variable position is thus identified by the codon number in the gene and base position in codon. Functional information provided by COG20.

Supplementary Table 10 – Filtered SCV tables at the inter-sample level for each metagenome, corresponding to the SNPs reported in Table S9. Table reports for each SCV: the unique identifier of the SCV (entry_id), unique position identifier of the SCV (unique_pos_identifier), name of the split where SCV is occurring (split_name), sample where the SCV has been identified (sample_id), unique gene caller identifier where the SCV is occurring (corresponding_gene_call with "-1" if the position is not in a gene call), order of the codon in the gene call (codon_order_in_gene with "-1" if the position is not in a gene call, codon_number), gene length (gene_length), number of recruited reads mapping to this codon (coverage), number of mapped reads covering the SCV corresponding to the different codons (64 codon combinations), reference codon in the reference genome (reference), most mapped codon (consensus), the two most represented codons (competing_codons), ratio of codon in a given SCV that diverge from the reference codon (departure_from_reference), ratio of codon in a given SCV that diverge from the consensus codon (departure_from_consensus), ratio of the second most frequent codon to the consensus codon (n2n1ratio), the entropy value (entropy)

and the synonymity values (pN, pS, nN, nS for consensus, reference and popular consensus). All these SCV information are also explained at the following link https://merenlab.org/2015/07/20/analyzing-variability/. Moreover, the table reports information related to gene clusters where the SCV occurs as in Table S6 (including the gene cluster id and the *w*SCG status of a gene) but also the prophage WO regions assignment (WO_assignmnent, WO_pct_alignment), *cid* genes assignment (cid_genes_from_Bonneau_et_al._2018 [6], *cid*_Bonneau_pct_alignment), the MLST and *wsp* genes assignment (MLST_and_wsp_genes_from_PubMLST [7] MLST_*wsp*_pct_alignment) and *w*SCG info (*w*SCG).

Supplementary Table 11 – Filtered SAAV tables at the inter-sample level for each metagenome, corresponding to the SNPs reported in Table S9. Table reports for each SAAV: the unique identifier of the SAAV (entry_id), unique position identifier of the SAAV (unique_pos_identifier), name of the split where SAAV is occurring (split_name), sample where the SAAV has been identified (sample_id), unique gene caller identifier where the SAAV is occurring (corresponding_gene_call with "-1" if the position is not in a gene call), order of the codon in the gene call (codon_order_in_gene with "-1" if the position is not in a gene call, codon_number), gene length (gene_length), number of recruited reads mapping to this amino acid (coverage), number of mapped reads covering the SAAV corresponding to the different amino acids (Ala, Arg, Asn, Asp, Cys, Gln, Glu, Gly, His, Ile, Leu, Lys, Met, Phe, Pro, STP, Ser, Thr, Trp, Tyr, Val), reference amino acid in the reference genome (reference), most mapped amino acid at the SAAV (consensus), the two most represented amino acids (competing_aas), ratio of amino acids in a given SAAV that diverge from the reference amino acids (departure_from_reference), ratio of amino acids in a given SAAV that diverge from the consensus amino acids (departure_from_consensus), ratio of the second most frequent amino acid to the consensus amino acid (n2n1ratio), the entropy value (entropy), the interchangeability of two amino acids represented by BLOSSUM (BLOcks SUbstitution Matrix) matrices (BLOSUM90, BLOSUM90_weighted, BLOSUM62, BLOSUM62_weighted). All these SAAV information are also explained at the following link https://merenlab.org/2015/07/20/analyzing-variability/. Moreover, the table reports information related to gene clusters where the SAAV occur as in Table S6 (including the gene cluster id and the *w*SCG status of a gene) but also the prophage WO regions assignment (WO_assignmnent, WO_pct_alignment), *cid* genes assignment (*cid*_genes_from_Bonneau_et_al._2018 [6], *cid*_Bonneau_pct_alignment), MLST and *wsp*

genes assignment (MLST_and_*wsp*_genes_from_PubMLST [7], MLST_*wsp*_pct_alignment) and *w*SCG information (*w*SCG).

Supplementary Table 12 - Number of SNPs (Single Nucleotide Polymorphisms), SCVs (Single Codon Variants) and SAAVs (Single Amino Acid Variants) identified for each MAG across the four metagenomes corresponding to different mosquito individuals, and SAAV/SCV ratio for each MAG.

Supplementary Table 13 – SNVs retained for manual inspection at the intra-sample level. Information reported as in Table S7 for the SNVs remaining within *w*SCGs after coverage outlier filtration and departure from consensus and entropy threshold.

Supplementary Figures

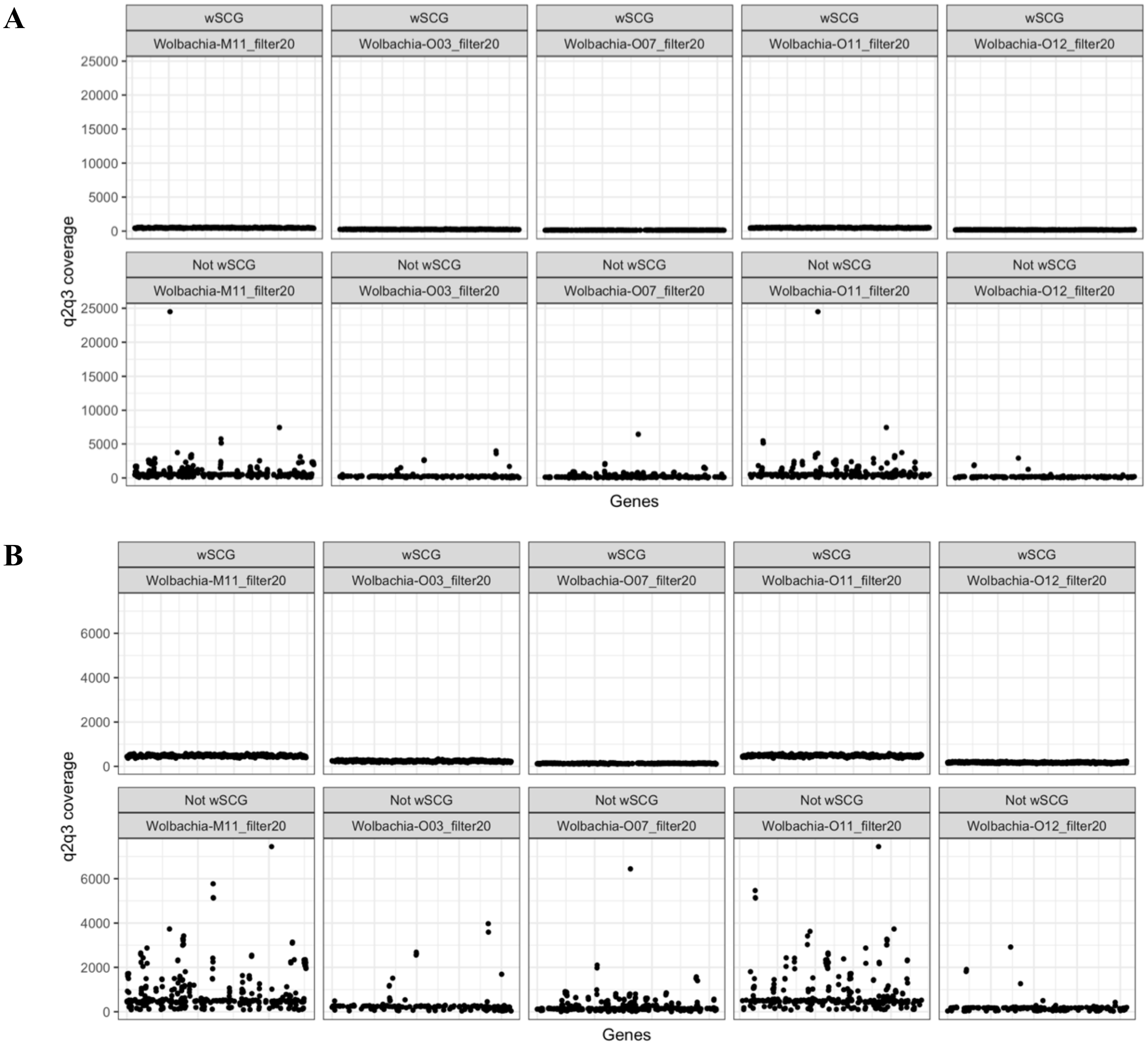

Supplementary Figure 1 – Reconstructed *Wolbachia* MAG gene coverage in the corresponding metagenome, depending on their status as *w*SCG or not. Metagenomic reads from each sample have been mapped to the corresponding *Wolbachia* MAG and coverage (q2_q3_cov) for each gene is represented on the y-axis. **A)** All genes. **B)** After removal of coverage outliers over 10,000X.

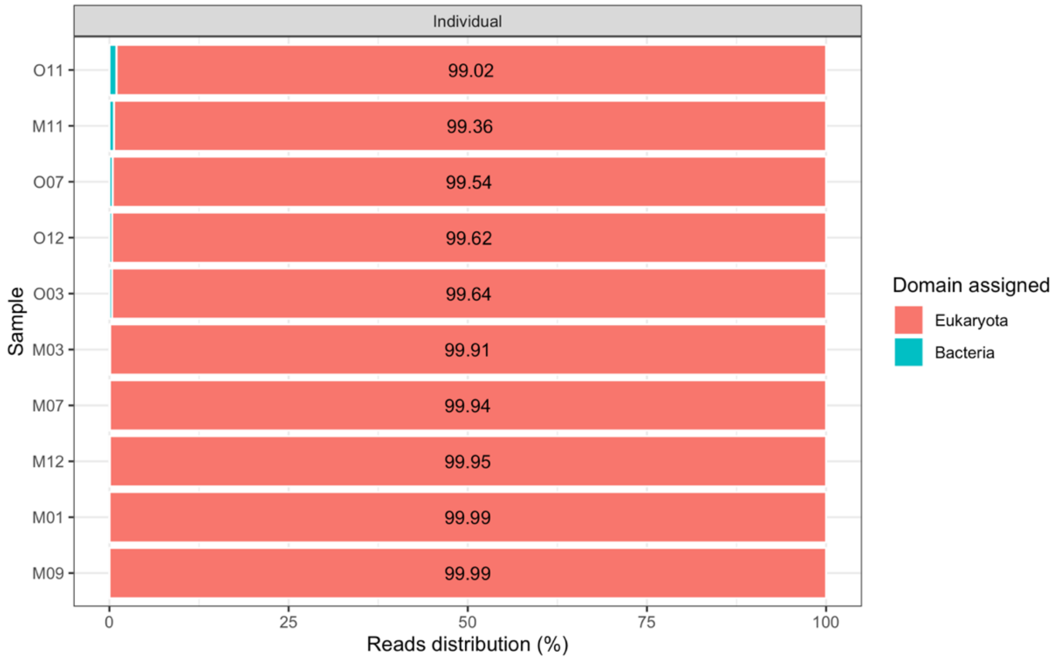

Supplementary Figure 2 – Estimation of the host contamination in our metagenomes based on phyloFlash (precisely from the taxonomic assignment of the mapped reads onto the SILVA SSU databases) showing a particularly high eukaryotic contamination rate in midgut samples that may explain the impossibility to reconstruct bacterial genomes in these samples. The red bar represents the proportion of eukaryotic reads detected in samples and the blue bar represents the bacterial percentage.

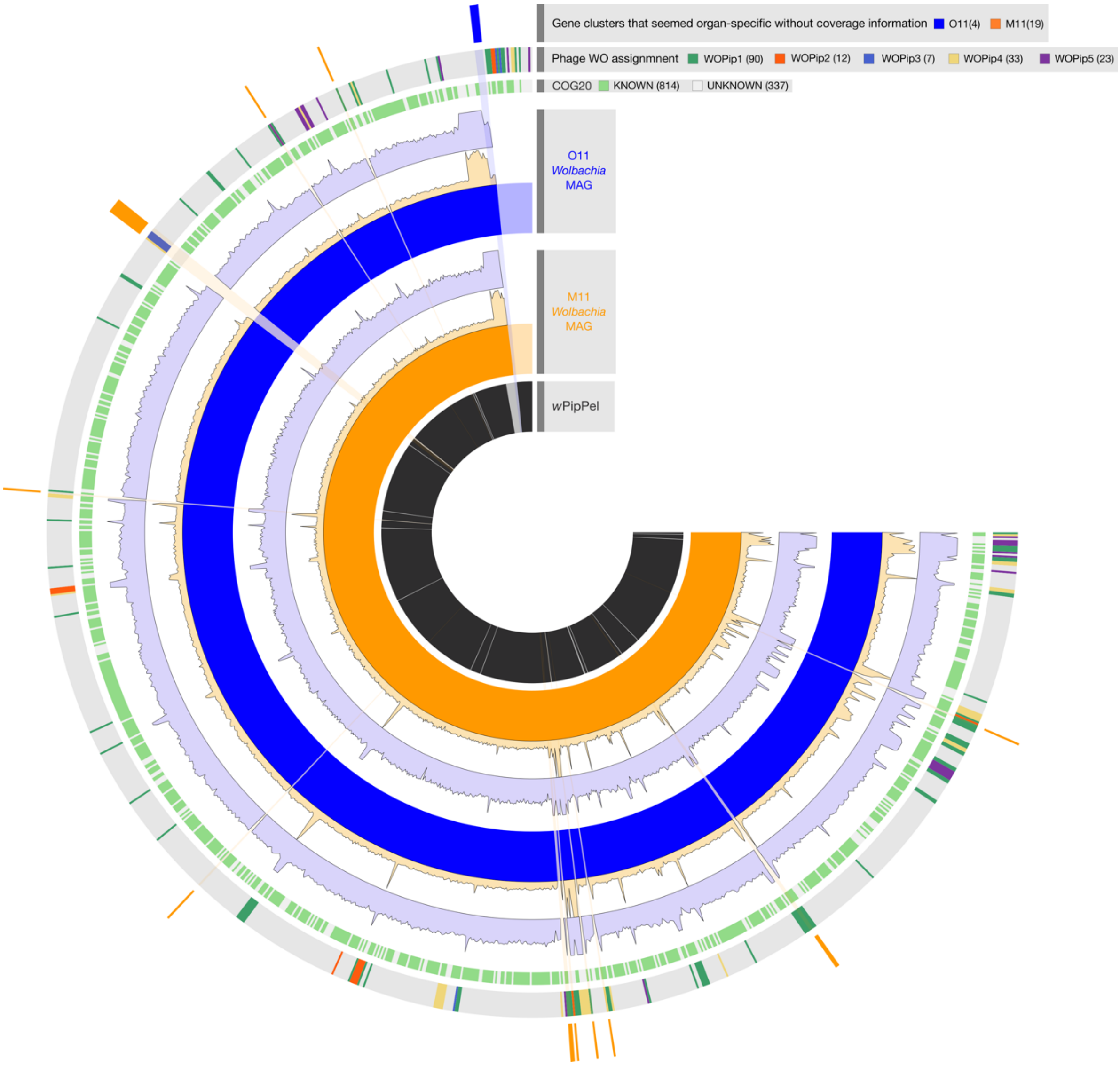

Supplementary Figure 3 –Metapangenome of *Wolbachia* MAG M11 (midgut sample) and MAG O11 (ovary sample) with gene clusters organized based on *Wolbachia* MAG M11 synteny because of its higher length compared to MAG O11. The black layer corresponds to the gene clusters from *Wolbachia* reference genome *w*PipPel. Information is gathered by groups of three layers: i) a gene cluster presence/absence layer (solid orange for MAG M11 and solid blue for MAG O11), ii) gene cluster mean coverage in sample M11 (light orange) and iii) gene cluster mean coverage in sample O11 (light blue). The maximum value for the coverage layers is set at 800X to improve their visualization. The three outer layers illustrate COG20 annotation status, the "phage-like" gene clusters identified from [8] in the *w*PipPel reference genome, and the gene clusters that seem to be present only in O11 or M11 *Wolbachia* MAGs.

263

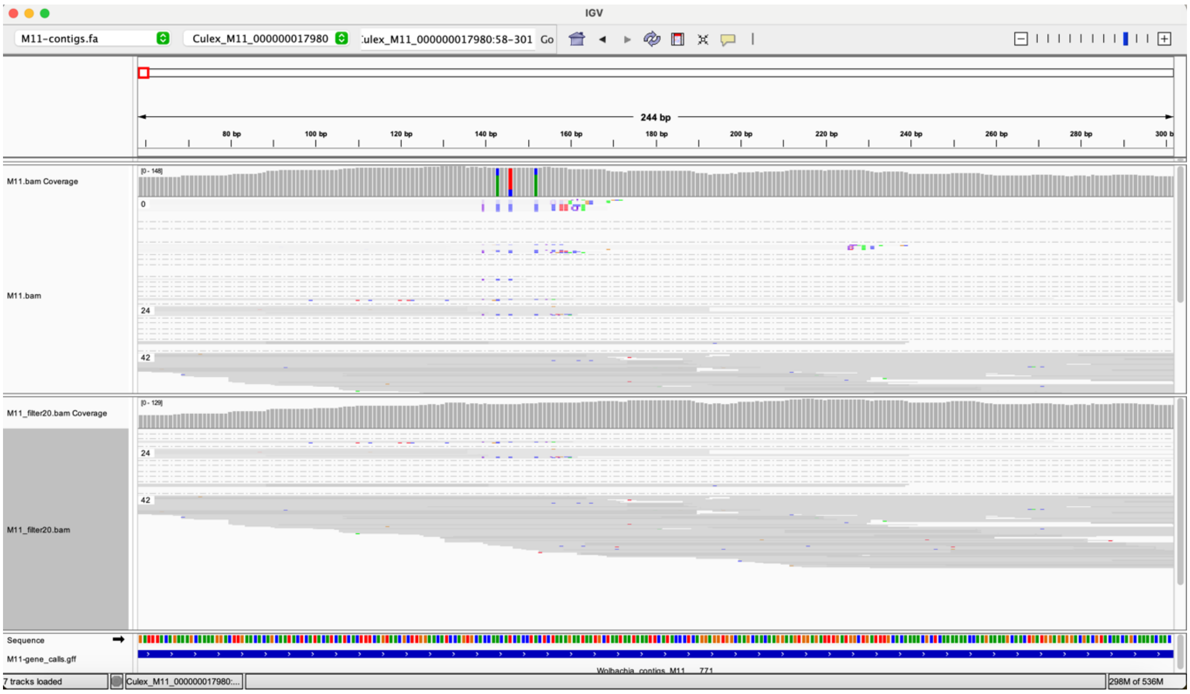

Supplementary Figure 4 – Visualization of contig 17980 from MAG M11, with raw mapping in the top part of the figure, and filtered mapping (MAPQ > 20) on the bottom. Screenshot from Integrative Genome Viewer (IGV).

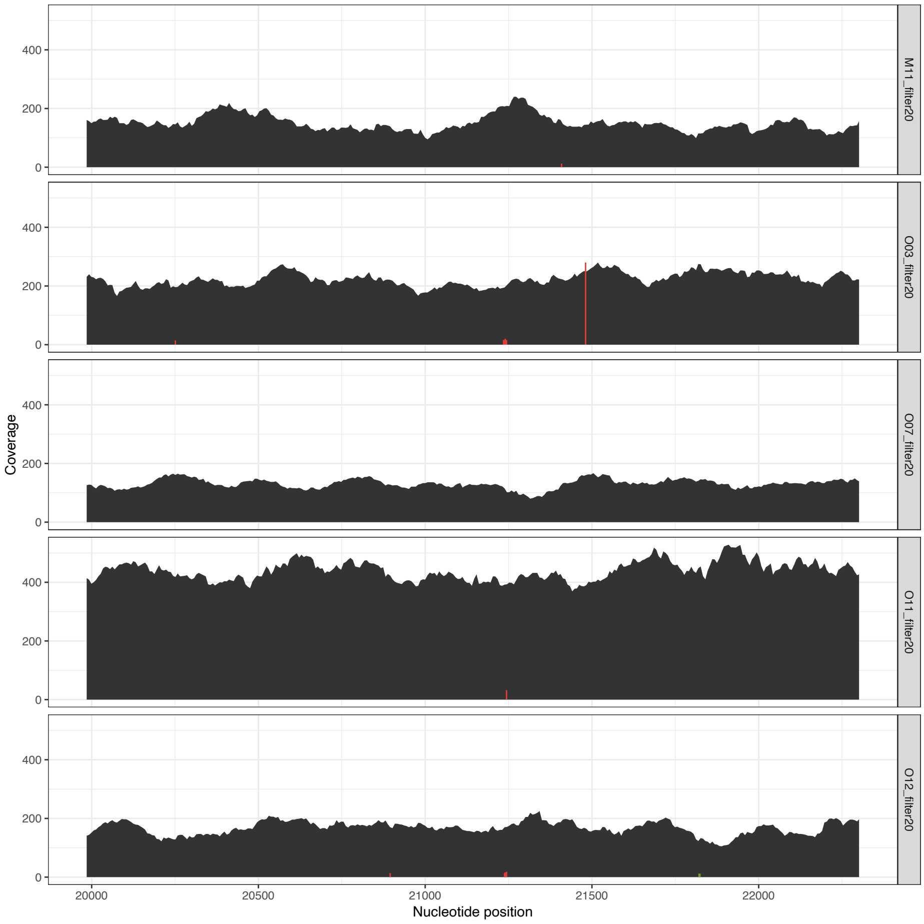

Supplementary Figure 5 – Visualization of gene 993 (gene cluster GC_00000983) from contig 8,388 in MAG O12. Black layers represent the gene's coverage in each metagenome. A SNP is identified in metagenome O03, in 2nd position in codon 501 (represented by a red bar). This gene was annotated as « translation initiation factor IF-2, a GTPase » by COG20.

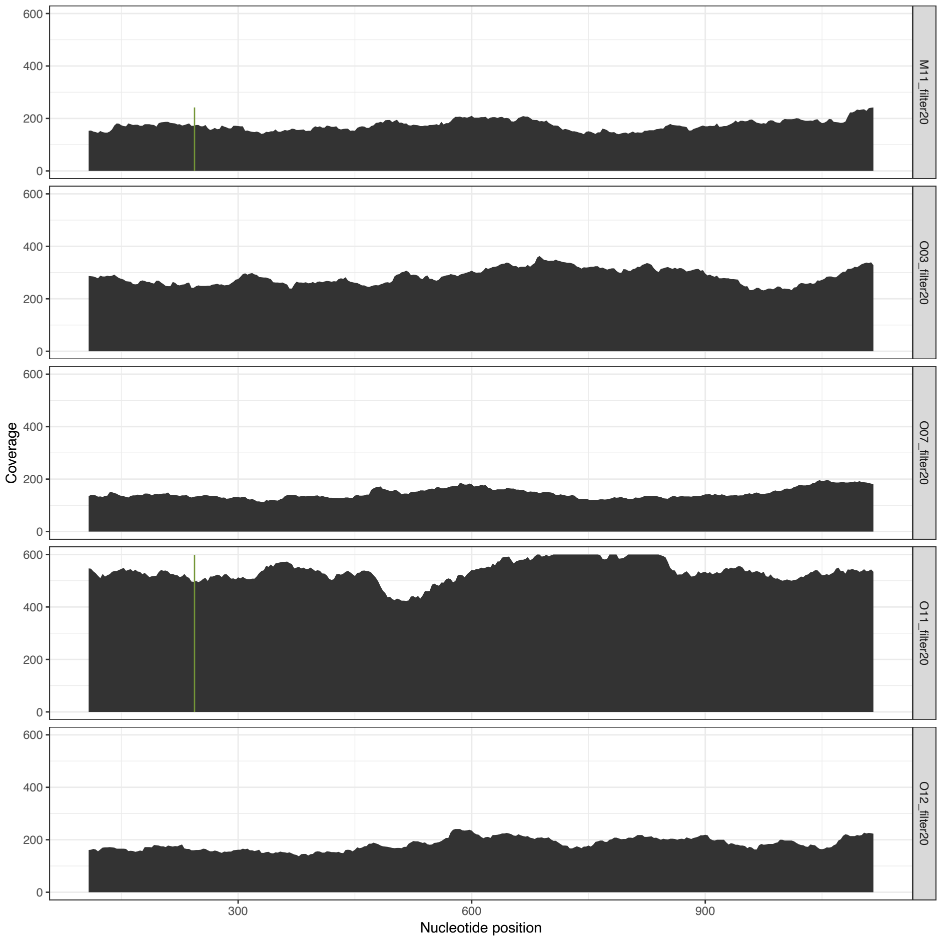

Supplementary Figure 6 – Visualization of gene 41 (gene cluster GC_00000685) from contig 68,699 split 2 in MAG O12. Black layers represent the gene's coverage in each metagenome. A SNP is identified in metagenomes O03 and O11, in 3rd position in codon 291 (represented by a green bar). This gene was annotated as « tRNA A37 threonylcarbamoyltransferase TsaD » by COG20.

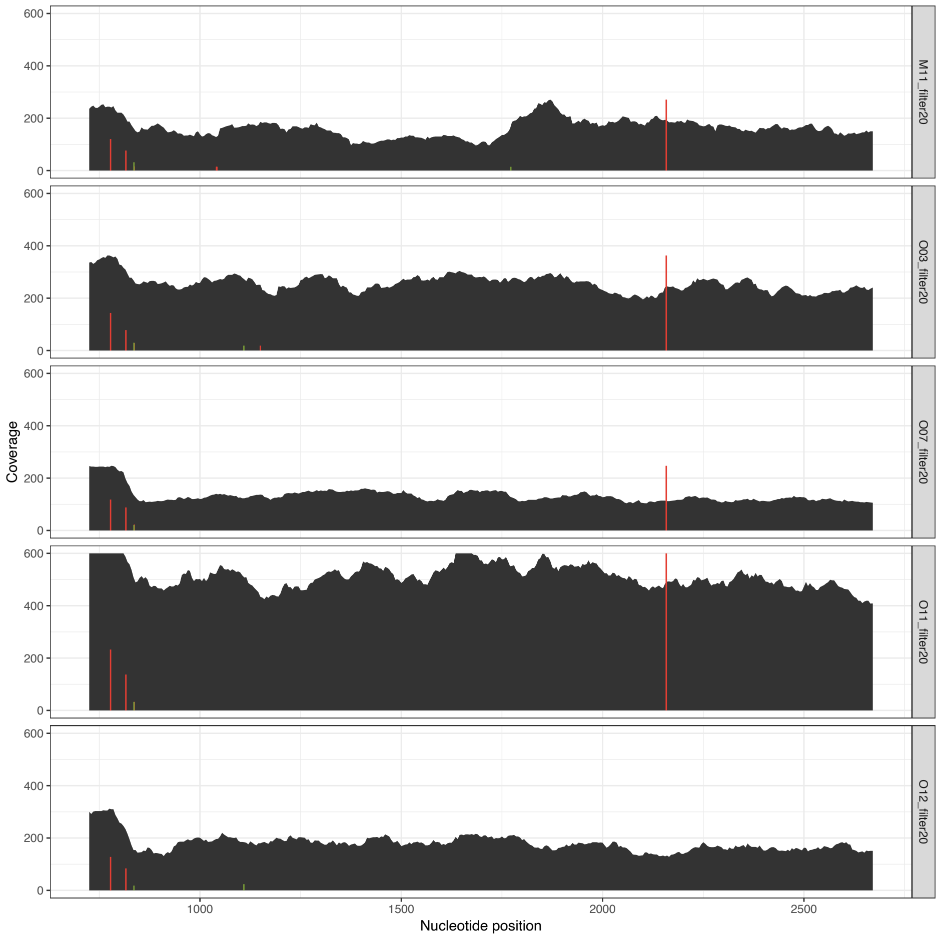

Supplementary Figure 7 – Visualization of gene 611 (gene cluster GC_00000967) from contig 221,252 in MAG O12. Black layers represent the gene’s coverage in each metagenome. A SNP is identified in metagenomes M11, O03, O07 and O11, in 1st position in codon 172 (represented by a red bar on the right-hand side of the figure). This gene was annotated as an « Ankyrin repeat » by COG20. On the left-hand side of the figure, some spurious SNVs are also visible. These were almost all flagged as coverage outliers.

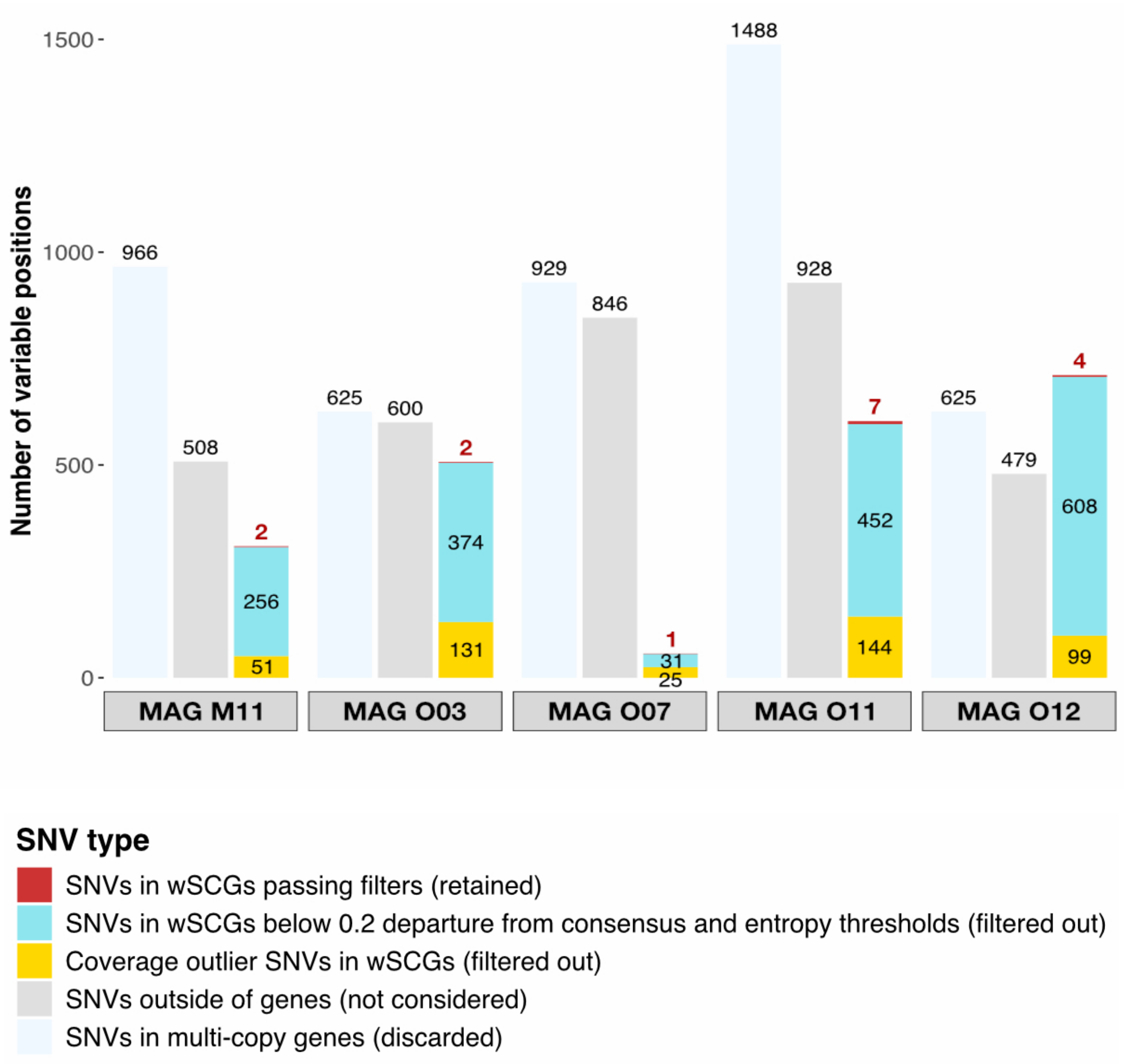

Supplementary Figure 8 – SNVs detected within samples for the five *Wolbachia* MAGs reconstructed. For each MAG, all SNVs identified by mapping the metagenome from which it was reconstructed are represented, with one bar for SNVs falling in multi-copy genes (left, clear blue), one bar for SNVs falling outside of genes (middle, grey), and one bar for SNVs falling in *w*SCGs (right). This third bar is subdivided between SNVs that were filtered out due to being coverage outliers (gold), SNVs with departure from consensus and entropy values below the 0.2 defined threshold (cyan), and finally SNVs that were retained (red).

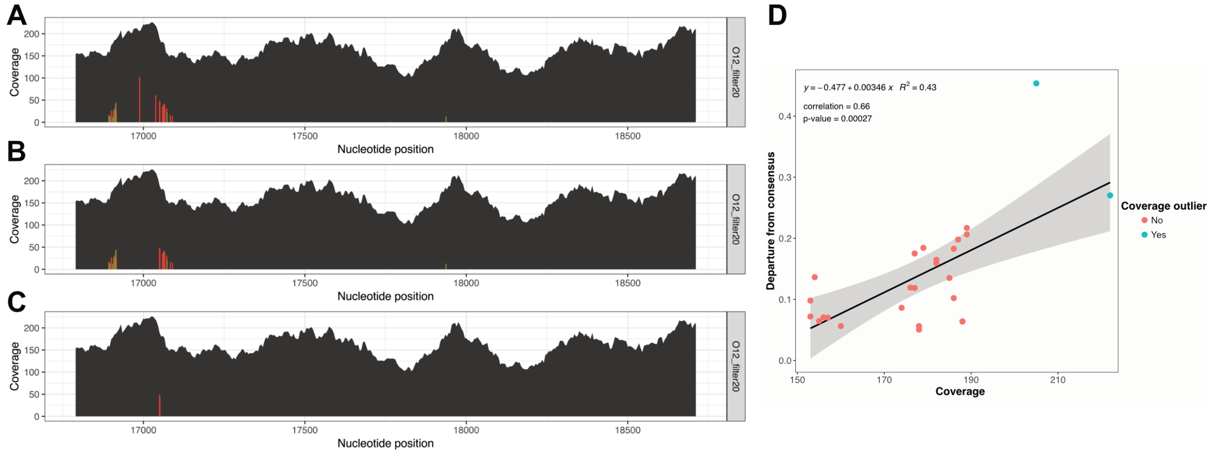

Supplementary Figure 9 – SNVs following recruitment of reads from O12 on *Wolbachia* MAG O12 for split 99075, gene 640. A) Raw SNVs occurring in gene 640 defined as *w*SCG annotated to an "ATP-dependent 26S proteasome regulatory subunit" by COG20 (Table S13). **B)** SNVs in gene 640 not identified as coverage outliers. **C)** SNVs in gene 640 passing the 0.2 departure from consensus and 0.2 entropy thresholds. For these three panels, the black layer represents the coverage in O12 and SNVs are represented by colored bars (green bars correspond to nucleotide difference in the 3rd position of the codon and red bars to nucleotide difference in the 1st or the 2nd one). The left y-axis corresponds to coverage values and the right y-axis corresponds to the variability values (departure from consensus). **D)** Correlation between departure from consensus and coverage values for all variable positions of gene 640 in MAG O12. The equation for the linear regression is noted in the top left corner of the plot, as well as the correlation coefficient and its p-value.

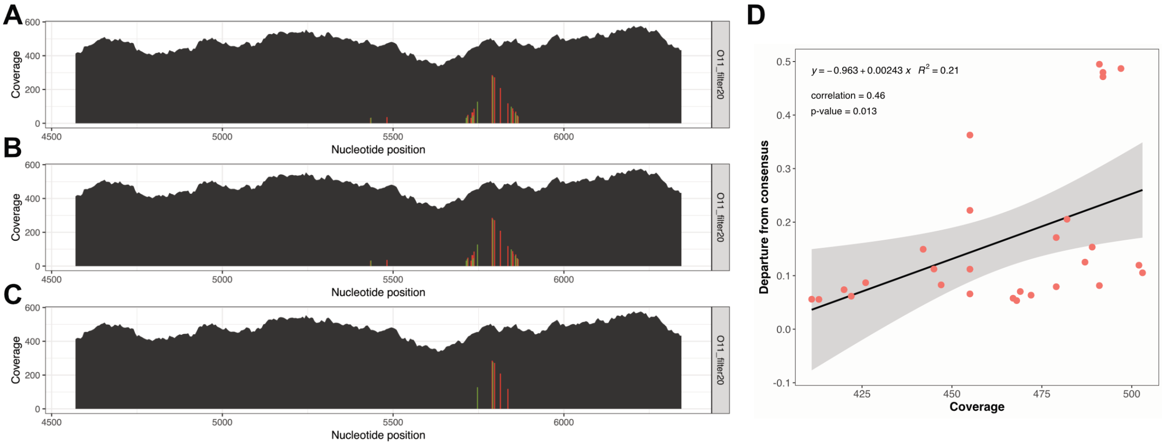

Supplementary Figure 10 – SNVs following recruitment of reads from O11 on *Wolbachia* MAG O11 for split 98617, gene 755. A) Raw SNVs occurring in gene 755 defined as *w*SCG annotated to an "Ankyrin repeat" by COG20 (Table S13). **B)** SNVs in gene 755 not identified as coverage outliers. **C)** SNVs in gene 755 passing the 0.2 departure from consensus and 0.2 entropy thresholds. For these three panels, the black layer represents the coverage in O11 and SNVs are represented by colored bars (green bars correspond to nucleotide difference in the 3rd position of the codon and red bars to nucleotide difference in the 1st or the 2nd one). The left y-axis corresponds to coverage values and the right y-axis corresponds to the variability values (departure from consensus). **D)** Correlation between departure from consensus and coverage values for all variable positions of gene 755 in MAG O11. The equation for the linear regression is noted in the top left corner of the plot, as well as the correlation coefficient and its p-value.

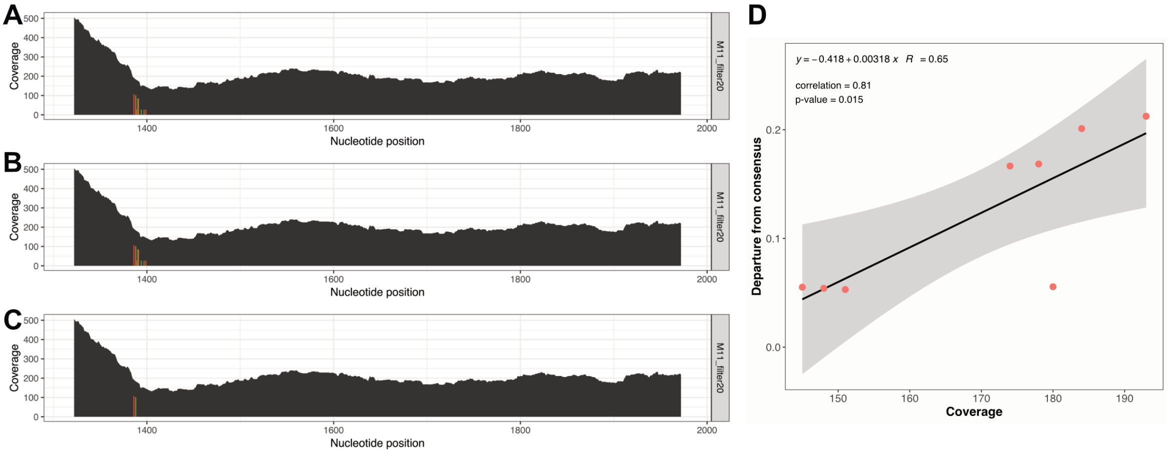

Supplementary Figure 11 – SNVs following recruitment of reads from M11 on *Wolbachia* MAG M11 for split 26618, gene 301. A) Raw SNVs occurring in gene 301 defined as *w*SCG and not annotated by COG20 (Table S13). **B)** SNVs in gene 301 not identified as coverage outliers. **C)** SNVs in gene 301 passing the 0.2 departure from

consensus and 0.2 entropy thresholds. For these three panels, the black layer represents the coverage in M11 and SNVs are represented by colored bars (green bars correspond to nucleotide difference in the 3rd position of the codon and red bars to nucleotide difference in the 1st or the 2nd one). The left y-axis corresponds to coverage values and the right y-axis corresponds to the variability values (departure from consensus). **D)** Correlation between departure from consensus and coverage values for all variable positions of gene 301 in MAG M11. The equation for the linear regression is noted in the top left corner of the plot, as well as the correlation coefficient and its p-value.

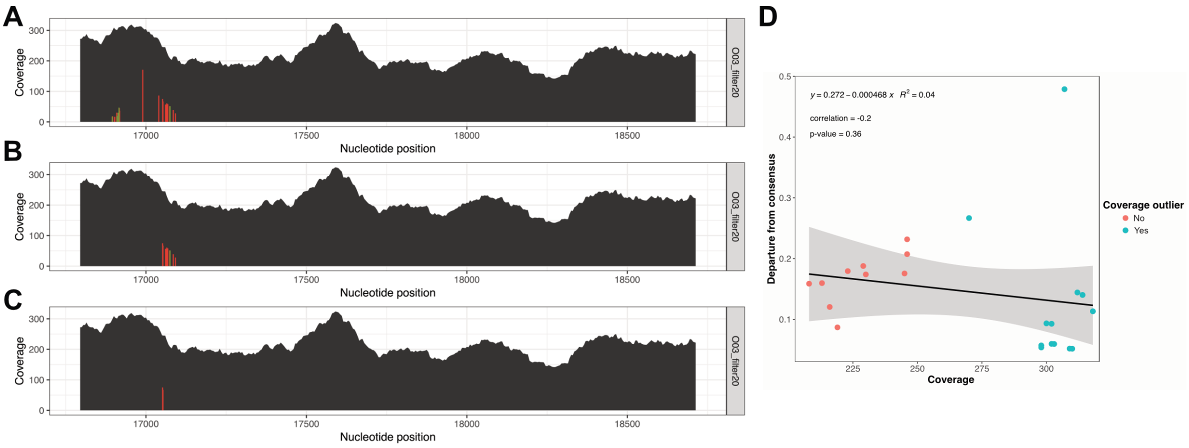

Supplementary Figure 12 – SNVs following recruitment of reads from O03 on *Wolbachia* MAG O03 for split 128791, gene 589. A) Raw SNVs occurring in gene 589 defined as *w*SCG annotated to an "ATP-dependent 26S proteasome regulatory subunit" by COG20 (Table S13). **B)** SNVs in gene 589 not identified as coverage outliers. **C)** SNVs in gene 589 passing the 0.2 departure from consensus and 0.2 entropy thresholds. For these three panels, the black layer represents the coverage in O03 and SNVs are represented by colored bars (green bars correspond to nucleotide difference in the 3rd position of the codon and red bars to nucleotide difference in the 1st or the 2nd one). The left y-axis corresponds to coverage values and the right y-axis corresponds to the variability values (departure from consensus). **D)** Correlation between departure from consensus and coverage values for all variable positions of gene 589 in MAG O03. The equation for the linear regression is noted in the top left corner of the plot, as well as the correlation coefficient and its p-value.

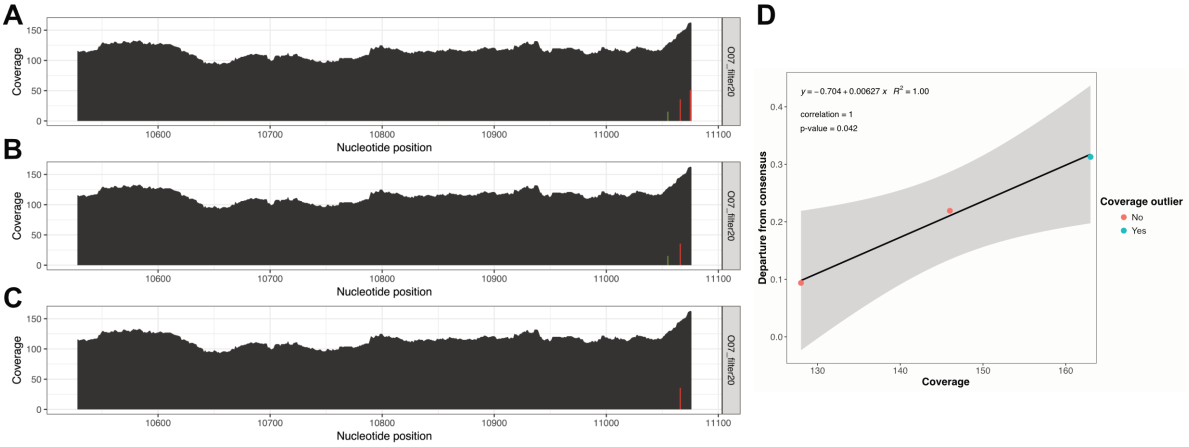

Supplementary Figure 13 – SNVs following recruitment of reads from O07 on *Wolbachia* MAG O07 for split 82262, gene 230. A) Raw SNVs occurring in gene 230 defined as *w*SCG, no annotated by COG20 (Table S13). **B)** SNVs in gene 230 not identified as coverage outliers. **C)** SNVs in gene 230 passing the 0.2 departure from consensus and 0.2 entropy thresholds. For these three panels, the black layer represents the coverage in O07 and SNVs are represented by colored bars (green bars correspond to nucleotide difference in the 3rd position of the codon and red bars to nucleotide difference in the 1st or the 2nd one). The left y-axis corresponds to coverage values and the right y-axis corresponds to the variability values (departure from consensus). **D)** Correlation between departure from consensus and coverage values for all variable positions of gene 230 in MAG O07. The equation for the linear regression is noted in the top left corner of the plot, as well as the correlation coefficient and its p-value.

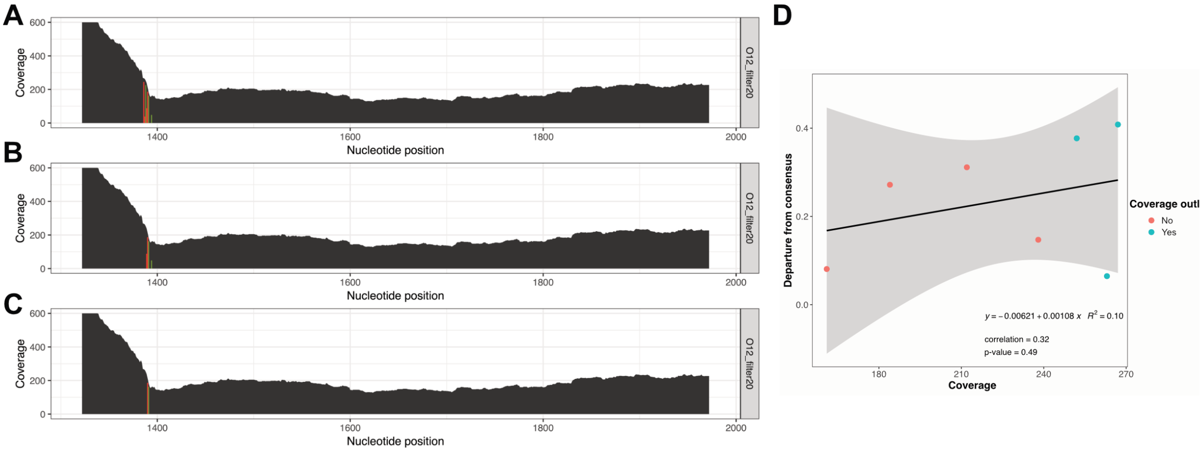

Supplementary Figure 14 – SNVs following recruitment of reads from O12 on *Wolbachia* MAG O12 for split 5804, gene 423. A) Raw SNVs occurring in gene 423 defined as *w*SCG, not annotated by COG20 (Table S13). **B)** SNVs in gene 423 not identified as

coverage outliers. **C)** SNVs in gene 423 passing the 0.2 departure from consensus and 0.2 entropy thresholds. For these three panels, the black layer represents the coverage in O12 and SNVs are represented by colored bars (green bars correspond to nucleotide difference in the 3rd position of the codon and red bars to nucleotide difference in the 1st or the 2nd one). The left y-axis corresponds to coverage values and the right y-axis corresponds to the variability values (departure from consensus). **D)** Correlation between departure from consensus and coverage values for all variable positions of gene 423 in MAG O12. The equation for the linear regression is noted in the top left corner of the plot, as well as the correlation coefficient and its p-value.

References

1. Alberti A, Poulain J, Engelen S, Labadie K, Romac S, Ferrera I, et al. Viral to metazoan marine plankton nucleotide sequences from the Tara Oceans expedition. *Sci Data* 2017; **4**: 170093.
2. Gruber-Vodicka HR, Seah BKB, Pruesse E. phyloFlash: Rapid Small-Subunit rRNA Profiling and Targeted Assembly from Metagenomes. *mSystems* 2020; **5**: e00920-20, /msystems/5/5/mSys.00920-20.atom.
3. Quast C, Pruesse E, Yilmaz P, Gerken J, Schweer T, Yarza P, et al. The SILVA ribosomal RNA gene database project: improved data processing and web-based tools. *Nucleic Acids Res* 2013; **41**: D590–D596.
4. Prjibelski A, Antipov D, Meleshko D, Lapidus A, Korobeynikov A. Using SPAdes De Novo Assembler. *Curr Protoc Bioinforma* 2020; **70**: e102.
5. R Core Team. R: The R Project for Statistical Computing. R version 3.6.3. https://www.r-project.org/. Accessed 7 Jun 2021.
6. Bonneau M, Atyame C, Beji M, Justy F, Cohen-Gonsaud M, Sicard M, et al. Culex pipiens crossing type diversity is governed by an amplified and polymorphic operon of Wolbachia. *Nat Commun* 2018; **9**: 319.
7. Jolley KA, Bray JE, Maiden MCJ. Open-access bacterial population genomics: BIGSdb software, the PubMLST.org website and their applications. *Wellcome Open Res* 2018; **3**: 124.
8. Klasson L, Walker T, Sebaihia M, Sanders MJ, Quail MA, Lord A, et al. Genome evolution of Wolbachia strain wPip from the Culex pipiens group. *Mol Biol Evol* 2008; **25**: 1877–1887.
